# Isolation and characterisation of *Klebsiella pneumoniae* bacteriophages vB_Kpn_Lilla1 and vB_Kpn_Lilla2

**DOI:** 10.1007/s00705-025-06374-7

**Published:** 2025-07-29

**Authors:** Timothy Martin, Liana Theodoridis, Emma Stent, Julia Lilla, Steve Petrovski

**Affiliations:** 1 Department of Microbiology, Anatomy, Physiology and Pharmacology, La Trobe University, Melbourne, VIC 3086 Australia; 2 La Trobe Institute for Molecular Science (LIMS), La Trobe University, Melbourne, VIC 3086 Australia

## Abstract

*Klebsiella pneumoniae* is an opportunistic pathogen that is commonly associated with pulmonary infections. It is of particular concern due to its increasing resistance to antibiotics, particularly carbapenem. Alternative treatments for these bacterial infections are required, and phage application is an attractive option. In this study, we isolated two novel *Klebsiella pneumonia* bacteriophages from wastewater, vB_Kpn_Lilla1 and vB_Kpn_Lilla2, both of which have lytic activity against *Klebsiella oxytoca* and *Klebsiella michiganensis*. Genome sequencing and phylogenetic analysis revealed that both phages belong to the family *Straboviridae*, with vB_Kpn_Lilla1 belonging to the genus *Jiaodavirus* and vB_Kpn_Lilla2 belonging to the genus *Slopekvirus*. Growth kinetics showed an effective reduction of *K. pneumoniae* populations when infected with either phage.

**Supplementary Information:**

The online version contains supplementary material available at 10.1007/s00705-025-06374-7.

## Introduction

*Klebsiella pneumoniae* is a Gram-negative bacterium that colonises the human gastrointestinal tract and nasopharynx [1, 2]. Although it is part of the native microbiota, *K. pneumoniae* can cause pneumonia, septicemia, and urinary tract infections and is a significant cause of nosocomial infections [3]. Moreover, *K. pneumoniae* is the most common pathogen causing neonatal infections, contributing to elevated mortality rates globally [4]. As a member of the ESKAPE pathogens, the prevalence of antibiotic resistance and carbapenem-resistant *K. pneumoniae* presents a global concern. Due to its resistance to most of the currently available antibiotics, new antimicrobial strategies are required for the treatment of these bacterial infections.

The use of bacteriophages has resurfaced as a potential alternative for treating clinical bacterial infections. To date, upwards of 1,196 *K. pneumoniae* phages have been identified; however, more phages with potential for use in phage therapy need to be isolated [5]. Expanding the library of *K. pneumoniae* phages provides a robust arsenal for alternative treatments, which is particularly crucial as antibiotics become increasingly ineffective against multidrug-resistant strains [6]. Expanding the phage repository can increase the potential for effective treatments against antibiotic-resistant bacterial pathogens [6].

In this study, we isolated and characterised two novel *K. pneumoniae* phages, vB_Kpn_Lilla1 and vB_Kpn_Lilla2, from wastewater. Both phages displayed efficient lytic activity *in vitro*, which warrants further investigation into their potential use to treat *K. pneumoniae* infections.

## Materials and methods

### Phage isolation and purification

Phages vB_Kpn_Lilla1 and vB_Kpn_Lilla2 were isolated from wastewater using the clinical *K. pneumoniae* isolate UQM2437. Briefly, wastewater samples were centrifuged at 10,000 *g* for 10 minutes, and the supernatant was passed through a 0.22-μm filter to remove bacterial cells. The filtrate was then used in phage overlay plates, which were visually inspected for plaques following an overnight incubation. Single plaques were purified at least three times to ensure the presence of a single phage. Plaque assays were performed using the double-layer overlay method to determine phage titers.

### Transmission electron microscopy

Copper grids (ProSciTech) coated with carbon and formvar were subjected to glow discharge treatment for 60 seconds. Five μL of high-titre phage filtrate was spotted onto the grid, and after 30 seconds, the excess residue was removed using filter paper. The samples were then negatively stained using 3 μL of 2% uranyl acetate, which was immediately removed with filter paper. The grid was left to dry for >30 minutes prior to visualisation using a JEM-2100 transmission electron microscope (JEOL).

### Phage DNA extraction, genome sequencing, annotation, and whole-genome analysis

To extract viral DNA, purified phage particles were precipitated using polyethylene glycol (PEG), followed by proteinase K treatment as described previously [7]. Isolated phage DNA (100 ng) was prepared using an NEBNext Ultra II FS DNA Library Prep Kit and sequenced using an Illumina MiSeq v2 300-cycle kit with 150-bp paired-end reads. Raw data were filtered using FASTp v0.23.4 [8], and the phage genome sequences were assembled using Unicycler v0.5.1 with default settings [9]. Open reading frames (ORFs) and tRNAs were predicted using Glimmer v3 in Geneious Prime 11.0.20.1 and Pharokka v1.3.2 [10]. Genes were annotated manually by comparing their predicted amino acid sequences with entries in the GenBank database.

For genomic similarity comparisons, the nucleotide sequences of vB_Kpn_Lilla1 and vB_Kpn_Lilla2 were analysed using VIRIDIC v1.0 (http://rhea.icbm.uni-oldenburg.de/VIRIDIC/). To assess genetic relatedness, the amino acid sequences of the encoded proteins were compared using vConTACT2 v0.11.3 [11]. A reticulate network was created using the vB_Kpn_Lilla1 and vB_Kpn_Lilla2 sequences together with all of the complete phage genome sequences in the GenBank database, using INPHARED (May 2025) [12]. The output network was visualised using Cytoscape v3.10.2 [13].

### Phage host range

The lytic activity of vB_Kpn_Lilla1 and vB_Kpn_Lilla2 was evaluated against 11 *K. pneumoniae*, seven *K. oxytoca*, and five *K. michiganensis* strains. Lawn plates of each *Klebsiella* strain were spotted with 10-µL aliquots of high-titre phage filtrate. The plates were incubated at 37 °C overnight, and viral plaques were observed. Phage lysis was scored as"complete","turbid", or"no lysis".

### Growth curves

To assess phage efficacy in coculture, a growth experiment was performed. Briefly, phages vB_Kpn_Lilla1 and vB_Kpn_Lilla2 were incubated independently with *K. pneumoniae* cultures (1.2 × 10^8^ CFU/mL) at a multiplicity of infection (MOI) of 0.05, 0.1, 0.5, or 1. Cocultures were incubated at 37ºC with shaking at 100 rpm. OD_600_ measurements were recorded every 30 minutes for 24 h using a CLARIOstar plate reader (BMG Labtech). Independent experiments were repeated in triplicate.

### Nucleotide sequence accession numbers

The nucleotide sequence of phages vB_Kpn_Lilla1 and vB_Kpn_Lilla2 were deposited in the GenBank database under the accession numbers PQ249162 and PQ249163, respectively.

## Results and discussion

### Bacteriophage isolation and morphology

**Fig. 1:**
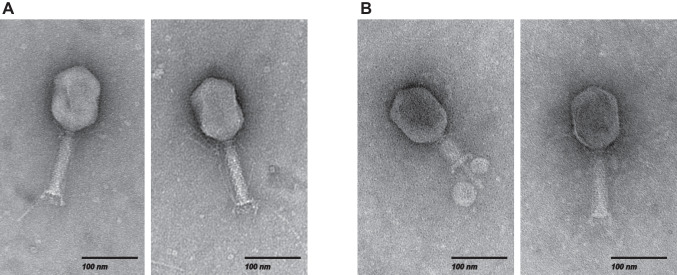
Transmission electron micrographs showing the morphology of *K. pneumoniae* phages Lilla1 (**A**) and Lilla2 (**B**). The scale bar is 100 nm

Two phages, vB_Kpn_Lilla1 and vB_Kpn_Lilla2 (henceforth called Lilla1 and Lilla2), were isolated from wastewater using *K. pneumoniae* isolate UQM2437 as the host. Both phages formed clear plaques after overnight incubation at 37ºC. Transmission electron microscopy revealed that both phages had myovirus morphology. The capsid of Lilla1 was found to have an average length of 120 nm and a width of 86 nm, while Lilla2 was found to have an average length of 116 nm and a width of 86 nm. The tail length was found to be 112 nm ± 5 nm for Lilla1 and 115 nm ± 4 nm for Lilla2. Long tail fibers were observed at the distal end of both phages (Fig. 1).

### Genome analysis of *K. pneumoniae* phages Lilla1 and Lilla2

**Fig. 2:**
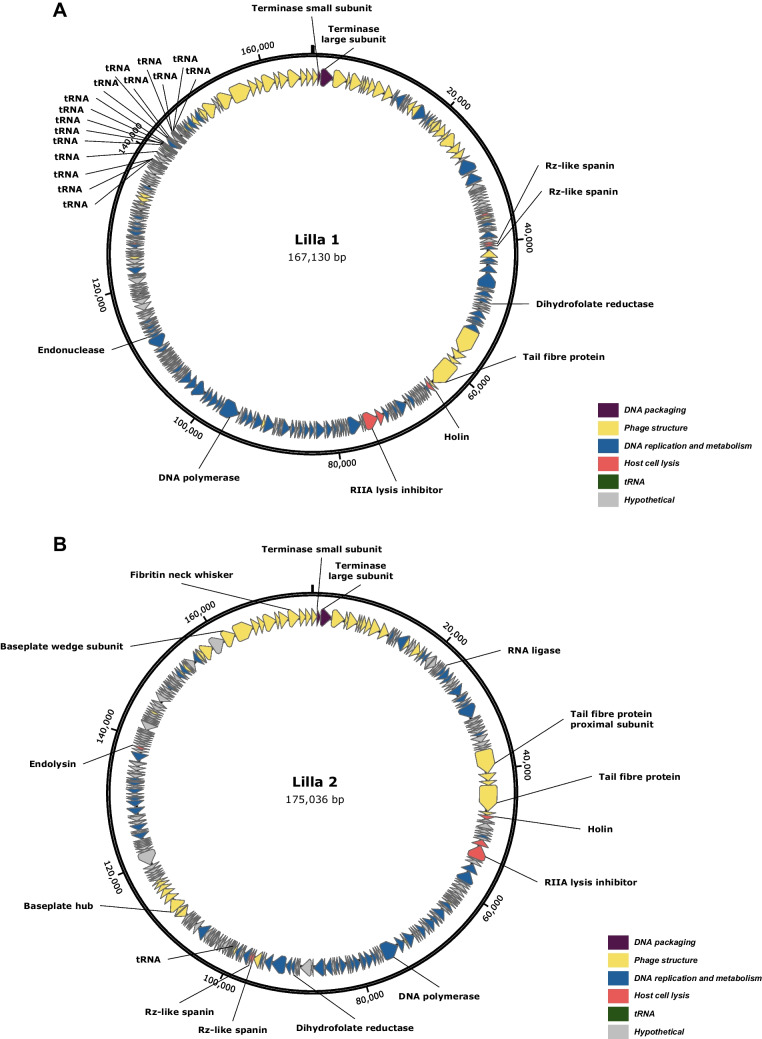
Circular genome maps of *K. pneumoniae* phages Lilla1 (**A**) and Lilla2 (**B**). Arrows represent the predicted ORFs and indicate the direction in which they are transcribed. Grey arrows represent ORFs encoding proteins with unknown functions. Coloured arrows indicate ORFs with assigned putative functions, as referenced in the associated colour key

Both *K. pneumoniae* phages were sequenced using an Illumina (MiSeq) platform and assembled using Unicycler v0.5.1. Proteins were identified using Pharokka v1.3.2 and confirmed using BLASTp analysis. Assembly of the Lilla1 and Lilla2 genome sequences showed that these phages have circular genomes of 167,130 bp and 175,036 bp, respectively, which is consistent with what is typically observed for myovirus phages [14]. Analysis of the genome of Lilla1 revealed a total of 298 ORFs, 148 (49.6%) of which were assigned predicted functions and 150 (50.4%) were annotated as hypothetical (Fig. 2A). Similarly, the genome of Lilla2 contains 283 ORFs, 163 of which (57.6%) were annotated as hypothetical (Fig. 2B). The genomes of Lilla1 and Lilla2 exhibit a modular arrangement, with three distinct head and tail morphogenesis modules, interspersed among other modular structures involved in DNA replication and metabolism, host cell lysis, genes of unknown function, and tRNA genes. A total of 16 tRNA genes were clustered in the Lilla1 genome, whereas only one was found in Lilla2. No genes associated with lysogeny were found in the genome of either phage, suggesting that they have a lytic lifestyle.

Lilla1 contains a holin gene (*orf93*) and two Rz-like spanin genes (*orf64-orf65*) but lacks a clearly identifiable endolysin gene. In contrast, Lilla2 encodes a holin (*orf63*), Rz-like spanins (*orf140-141*), and a predicted endolysin (*orf228*), consistent with a canonical lytic module. Given the presence of holin and spanin components in Lilla1, it is plausible that one of its genes of currently unknown function may encode an endolysin, potentially representing a novel or highly divergent variant that was not readily detected by the standard annotation tools that were used.

Although no known virulence factors or lysogeny-related genes were identified, both genomes encode a dihydrofolate reductase (DHFR) gene, which is associated with trimethoprim resistance [15]. Although DHFR genes have been identified previously in phage genomes and this enzyme might have metabolic functions, its role in antimicrobial resistance should be evaluated when considering these phages for any applications. In addition to genes that encode structural proteins, phage DNA packaging enzymes, and proteins involved in host cell lysis and DNA replication and metabolism, many other genes encode products whose functions are currently unknown based on existing annotation tools.

### Phylogenetic and taxonomic analysis and host range

**Fig. 3:**
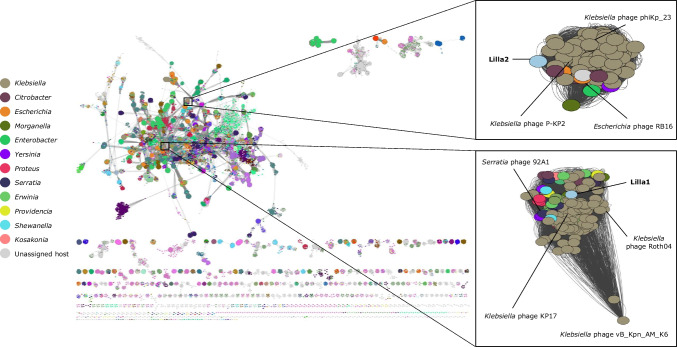
Network phylogeny of the Lilla1 and Lilla2 phage genomes. A reticulate network of the genome sequences of Lilla1 and Lilla2 and over 23,000 phages (nodes, represented as circles) with sequences in the GenBank database is shown, with key bacterial host genera colour coded to highlight their placement. The connecting lines (edges) indicate relatedness based on shared proteins. Shorter edges reflect stronger similarities. The global network is annotated by phage family, and the zoomed Lilla clusters are colour-coded by bacterial host genus

**Fig. 4:**
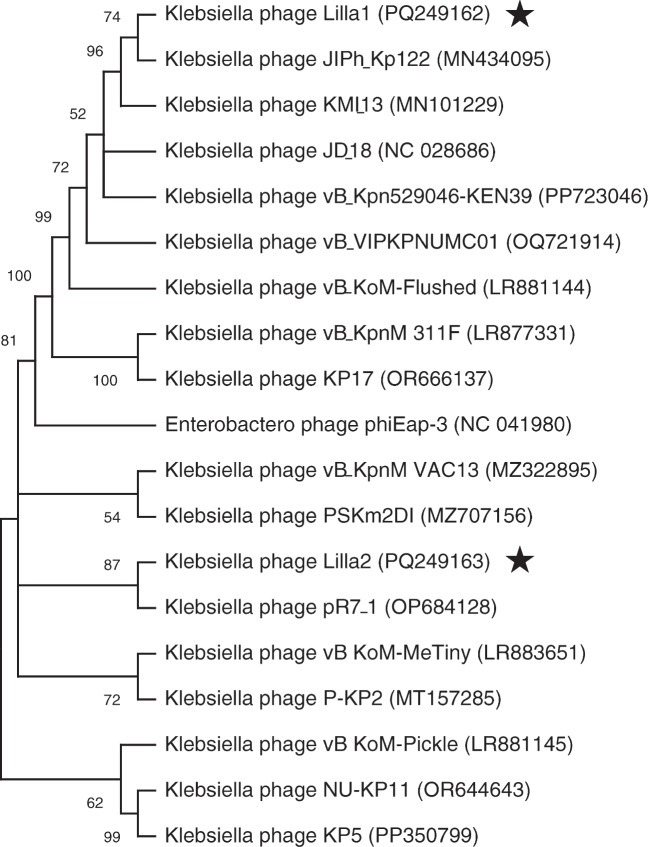
Phylogenetic relationships of *K. pneumoniae* phages and their top GenBank homologues. Phylogenetic trees for Lilla1 and Lilla2 (starred) were constructed using alignments of terminase large subunit sequences. Bootstrap values are shown next to the branches

To identify genes that Lilla1 and Lilla2 have in common with other phages, a vConTACT2 reticulate network was generated using the INPHARED reference database. The global network included >31,000 phage genome sequences from the GenBank database as of May 2025 and were colour-coded by host genus (Fig. 3). Both Lilla1 and Lilla2 were found to cluster within the main network with phages belonging to the family *Straboviridae*, confirming their taxonomic classification at the family level. First-joining neighbour clusters were expanded, showing that the proteins encoded by Lilla1 had high amino acid sequence similarity to those of 187 phages infecting members of the genera *Klebsiella*, *Erwinia*, *Shewanella*, *Yersinia*, and *Serratia*. Lilla2 clustered with 127 phages and displayed genetic similarity to phages infecting members of the genera *Klebsiella*, *Escherichia*, *Citrobacter*, and *Enterobacter.* To examine the evolutionary relationships of the Lilla phages, a phylogenetic tree was constructed based on sequences of the large subunit of the phage terminase. As expected, both Lilla1 and Lilla2 showed a strong evolutionary relationship to other *Klebsiella*-infecting phages within the family *Straboviridae*, and this was supported by high bootstrap values (Fig. 4).

**Fig. 5:**
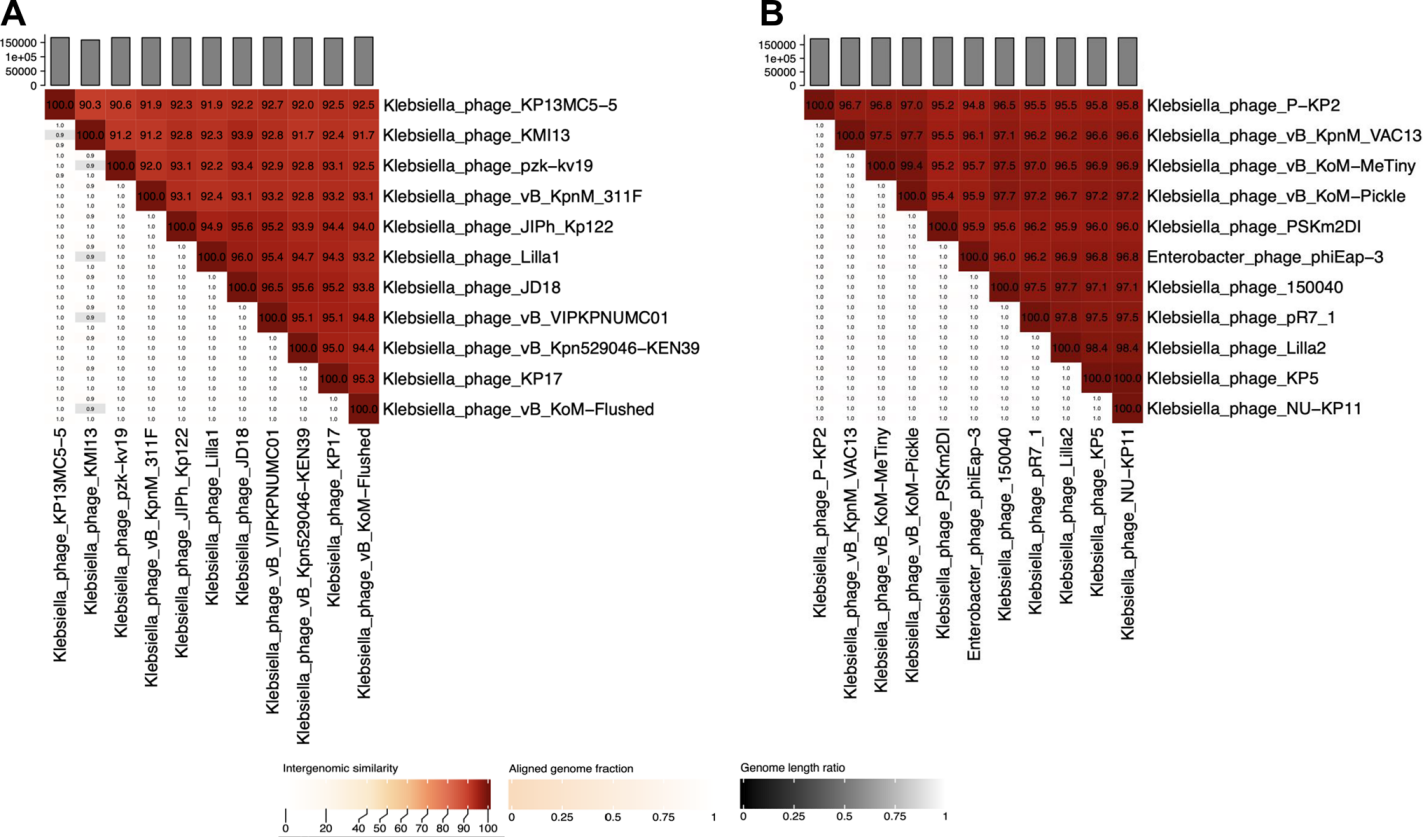
Intergenomic similarity heat maps of phages Lilla1 (**A**) and Lilla2 (**B**) based on VIRIDIC analysis of these phages and their respective closest relatives. The percentage of similarity is shown on the right, and aligned genome fraction is shown on the left. The genome size of each phage is shown in the graph at the top

To identify intergenomic similarities, VIRIDIC analysis was performed using sequences from the two isolated *K. pneumoniae* phages and their homologues obtained from the GenBank database. Comparing Lilla1 with the 10 most closely related genomes highlighted strong intergenomic similarity, with identity values ranging from 91.9% (Lilla1 vs. KP13MC5-5) to 96% (Lilla1 vs. JD18) (Fig. 5A). Similarly, Lilla2 comparisons displayed similarity to other phage sequences, with identity values ranging from 95.5% (Lilla 2 vs. P-KP2) to 98.4% (Lilla2 vs. KP5 and NU-KP11) (Fig. 5B). Because all of these values are above the threshold of 70% identity for demarcation of genera [16], Lilla1 and Lilla2 should be classified as members of the genera *Jiaodavirus* and *Slopekvirus*, respectively. Phages belonging to these genera have been shown to exhibit a broad host range [17, 18].

Lilla1 and Lilla2 both exhibited lytic activity against a diverse panel of clinical *Klebsiella* strains. Both lysed multiple clinical isolates of *K. pneumoniae*, including bloodstream and respiratory samples, with Lilla1 demonstrating a broader host range than Lilla2. Lilla1 showed lysis in 20 out of 23 *Klebsiella* isolates tested, including strains of *K. pneumoniae*, *K. oxytoca*, and *K. michiganensis* (Table 1). Lilla2 displayed activity against eight isolates, with strong lysis in some *K. pneumoniae* and *K. oxytoca* strains. Neither phage lysed non-*Klebsiella* species such as *E. coli* or *Salmonella* Typhimurium, suggesting genus-level specificity. These results show that Lilla1 has a broad host range within the genus *Klebsiella*, like other members of the genus *Jiaodavirus*. The narrower spectrum observed for Lilla2 may reflect greater receptor specificity.

**Table 1:** Host range of *Klebsiella pneumoniae* bacteriophages on *Klebsiella* isolates

*S*train			Lysis	
Isolate name	Source	Year isolated	Lilla 1	Lilla 2
*Klebsiella pneumoniae* UQM2437	NA	NA	++	++
*Klebsiella pneumoniae* C1	Blood	2025	+	-
*Klebsiella pneumoniae* C2	Blood	2025	++	-
*Klebsiella pneumoniae* C3	Blood	2025	++	+
*Klebsiella pneumoniae* C4	Blood	2025	+	-
*Klebsiella pneumoniae* C5	Blood	2025	++	+
*Klebsiella pneumoniae* VMS01	Blood	2025	+	+
*Klebsiella pneumoniae* VMS02	Blood	2025	-	-
*Klebsiella pneumoniae* VMS03	Blood	2025	++	++
*Klebsiella pneumoniae* VMS04	Blood	2025	+	-
*Klebsiella pneumoniae* 7267	Respiratory	2017	-	-
*Klebsiella pneumoniae subsp. ozaenae* 0049	Wound	2018	-	-
*Klebsiella oxytoca* 4024	Faeces	2017	+	++
*Klebsiella oxytoca* 6152	Faeces	2017	+	+
*Klebsiella oxytoca* 7873	Faeces	2017	+	-
*Klebsiella oxytoca* 9778	Faeces	2017	++	-
*Klebsiella oxytoca* 0478	Faeces	2017	+	-
*Klebsiella oxytoca* 0478	Faeces	2017	+	-
*Klebsiella oxytoca* 4300	Faeces	2019	++	-
*Klebsiella michiganensis* 818	Faeces	2017	++	-
*Klebsiella michiganensis* 727	Faeces	2017	+	++
*Klebsiella michiganensis* 735	Faeces	2017	++	-
*Klebsiella michiganensis* 0471	Faeces	2017	+	+
*Klebsiella michiganensis* 0503	Faeces	2017	+	-

For spot testing, ++ indicates complete lysis, + indicates turbid lysis, and - indicates no lysis

### Growth dynamics of *K. pneumoniae* in coculture with Lilla phages

**Fig. 6:**
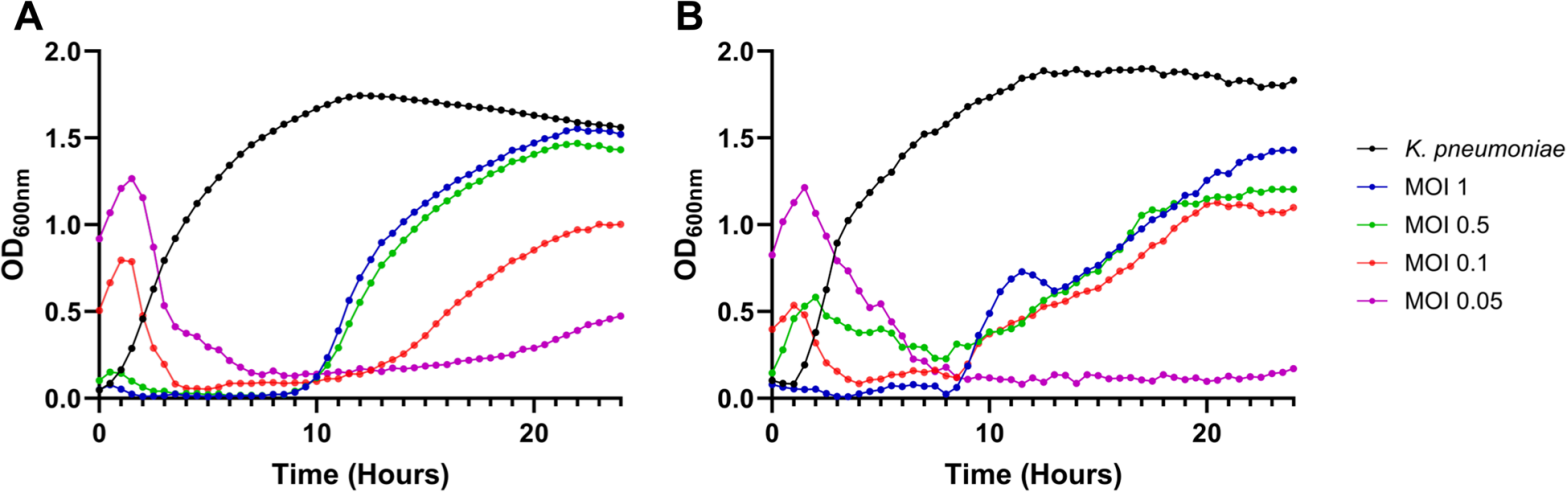
Growth curve of *K. pneumoniae* and phage Lilla1 (**A**) and Lilla2 (**B**) coculture. The phages were cocultured with *K. pneumoniae* (OD_600_ = 0.05) at an MOI of 1 (blue), 0.5 (green), 0.1 (red), or 0.05 (purple) for 24 h. An uninfected *K. pneumoniae* monoculture (black) was used as a control. The data are presented as the mean ± STD (error bars) from two independent biological replicates, with individual data points represented by closed circles

Growth curve analysis was conducted using different starting concentrations of *K. pneumoniae* and different MOIs. This was done to test the ability of the Lilla phages to eliminate their bacterial hosts at different optical densities. The bacterial cultures initially continued to grow, and this was presumably the stage in which the phages were being absorbed. In each experiment, a dramatic decrease in the growth of *K. pneumoniae* was observed within 60 minutes (Fig. 6). At an MOI of 1 or 0.5, *K. pneumoniae* growth was suppressed for up to 10 h. At a reduced MOI of 0.05, bacterial regrowth was delayed until approximately 20 h post-inoculation. This demonstrated that a lower phage MOI can suppress *K. pneumoniae* growth for an extended period of time. At an MOI of 0.05, robust lytic activity and a delay in the development of resistance were observed, suggesting that this is the optimal dosage for clearing the bacterial host. Similar findings were reported previously for *Burkholderia pseudomallei* phage vB_BpP_HN01, for which an MOI of 0.1 was demonstrated to be optimal for antibacterial activity [19]. It has been shown previously that diverse bacterial populations and nutrient competition *in vivo* can enhance phage efficacy despite the observation of the development of resistance *in vitro* [20].

To better understand the infection mechanisms of Lilla1 and Lilla2, future studies should focus on the identification of bacterial receptors. Moreover, testing these phages using murine models *in vivo* will provide critical insights into their safety and effectiveness as treatments for multidrug-resistant *K. pneumoniae* infections.

## Supplementary Information

Below is the link to the electronic supplementary material.

Supplementary file1 (GB 356 KB)

Supplementary file2 (GB 363 KB)
